# Closed chamber globe stabilization and needle capsulorhexis using irrigation hand piece of bimanual irrigation and aspiration system

**DOI:** 10.1186/1471-2415-5-21

**Published:** 2005-08-18

**Authors:** Harinder S Sethi, Tanuj Dada, Harminder K Rai, Prabhpreet Sethi

**Affiliations:** 1 Rajendra Prasad Centre for Ophthalmic Sciences, All India Institute of Medical Sciences, New Delhi 110029, India; 2 Lala Ram Saroop Institute, Mehrauli, New Delhi, India

## Abstract

**Background:**

The prerequisites for a good capsulorhexis include a deep, well maintained anterior chamber, globe stabilization and globe manipulation. This helps to achieve a capsulorhexis of optimal size, shape and obtain the best possible position for a red glow under retroillumination. We report the use of irrigation handpiece of bimanual irrigation aspiration system to stabilize the globe, maintain a deep anterior chamber and manipulate the globe to a position of optimal red reflex during needle capsulorhexis in phacoemulsification.

**Methods:**

Two side ports are made with 20 G MVR 'V' lance knife (Alcon, USA). The irrigation handpiece with irrigation on is introduced into the anterior chamber through one side port and the 26-G cystitome (made from 26-G needle) is introduced through the other. The capsolurhexis is completed with the needle.

**Results:**

Needle capsulorhexis with this technique was used in 30 cases of uncomplicated immature senile cataracts. 10 cases were done under peribulbar anaesthesia and 20 under topical anaesthesia. A complete capsulorhexis was achieved in all cases.

**Conclusion:**

The irrigating handpiece maintains deep anterior chamber, stabilizes the globe, facilitates pupillary dilatation, and helps in maintaining the eye in the position with optimal red reflex during needle capsulorhexis. This technique is a safe and effective way to perform needle capsulorhexis.

## Background

The anterior capsulorhexis has got several intra and post operative advantages over can opener or endocapsular capsulotomies and has become the standard capsulotomy technique for phacoemulsification [1-3]. Anterior capsulorhexis can be performed using 26 G bent needle cystitome or Utratas forceps [1,2]. The needle capsulorhexis can be performed through side port incision using a viscoelastic device or an anterior chamber maintainer [1,2,4,5]. During the performance of capsulorhexis, the globe can be stabilized either using a second instrument such as a Sinskey hook, or by holding limbal conjunctiva with a Lim's forceps. Sinskey hook introduced through a separate side port incision can lead to egress of viscoelastic from the eye and hence risk of radial extension of capsular flap. Holding conjunctiva with Lim's forceps can be traumatic or undesirable under topical anaesthesia.

The prerequisites for a good capsulorhexis include a deep well maintained anterior chamber, globe stabilization and globe manipulation to achieve best position for a red glow under retroillumination. All these can be achieved by the technique described by us, which is a modification of anterior capsulorhexis under anterior chamber maintainer described previously by Blumenthal [4].

## Methods

**Figure 1:**
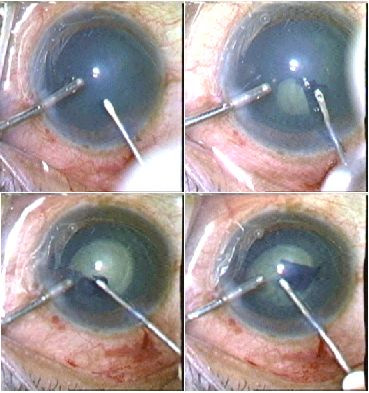
Closed chamber globe stabilization with irrigation hand piece and needle capsulorhexis with 26 G cystitome.

Two side ports are made with 20 G V lance knife (Alcon, Fort Worth Texas, USA) at 10 'O' clock and 2 'O' clock. A bent cystitome is made from 26-G needle. The irrigation hand piece of Bimanual irrigation aspiration system (Appasamy Associates, Chennai, India) is introduced into the anterior chamber through 2 'O' Clock side port with irrigation on. The irrigation hand piece is attached to balanced salt solution with bottle height of 90 – 95 cm above eye level. The 26-G cystitome is introduced through 10 'O' clock side port. The relaxing incision is made in the central area of anterior capsule, and a flap is created. This flap is flipped and reflected upon the underlying capsule so that epithelial side now faces the cornea. The reflected flap is then engaged near the tearing edge and rotated in a circular manner. When almost entire circumference of the central capsular opening is achieved, the flap is pulled centripetally inwards. This joins the capsular edges from outside in to complete the capsulorhexis (Figure 1). During this period, the irrigation hand piece held in the left hand maintains deep anterior chamber and stabilizes the globe. It also helps to keep globe in the best position for a good red glow.

## Results

We have used this technique in 30 cases of uncomplicated immature senile cataracts. The cases with intumuscent cataracts, white cataracts, corneal opacities or other ocular pathologies were not included.10 cases were done under peribulbar anaesthesia and 20 under topical anaesthesia. A complete capsulorhexis was achieved in all cases.

## Discussion

The anterior capsulorhexis is one of the prerequisites for successful and uncomplicated phacoemulsification [1]. The needle capsulorhexis can be performed under an anterior chamber maintainer, which requires an extra side port incision inferiorly, whereas in our technique the side port made for the second instrument is used for introducing the irrigation handpiece. In addition one needs to stabilize the globe using a Lim's forceps or Sinskey hook introduced through side port. The Sinskey hook introduced through side port can lead to egress of viscoelastic or fluid, which can lead to shallowing of the anterior chamber. Holding conjunctiva with lims's forceps can lead to hemorrhage, tear and increased patient discomfort. This is undesirable, especially under topical anaesthesia. These problems can be overcome by using the above described technique.

We prefer to use irrigation hand piece to achieve well maintained deep anterior chamber and relaxed zonules throughout the capsulorhexis. In addition, the irrigation handpiece snuggly fits into the side port incision minimizing the fluid leaks. This also helps in moving the globe to an optimal position for the best red glow under retroillumination. The slight manipulation of the globe in a direction opposite to the needle movement facilitates the rotation of the anterior capsular flap with needle. Our technique is especially useful for the phacoemulsificaton under topical anaesthesia, where holding the conjunctiva for globe rotation is undesirable and can be painful for the patient. This can also cause conjunctival tear and subconjunctival hemorrhage. Our technique has a special role in the developing countries where reducing the amount of viscoelastic used can decrease the overall cost of surgery.

With continuous irrigation, the capsular flap floats freely in the anterior chamber. This improves visualization and maneuverability. This increased mobility of the anterior capsular flap may be troublesome for the beginners. It can be minimized by keeping the direction of flow of fluid away from the site of flap rotation. The capsulorhexis under the irrigation fluid demands a tight incision and undue posterior pressure on these incisions is undesirable as this may lead to sudden egress of fluid and the flap may extend radially outwards [2].

The described technique by us is simple and needs reapplication of already described techniques, hence can be easily mastered by all surgeons.

## Conclusion

The irrigating handpiece maintains deep anterior chamber, stabilizes the globe, facilitates pupillary dilatation, helps in maintaining the eye in the position with optimal red reflex during needle capsulorhexis. This technique is a safe and effective way to perform needle capsulorhexis.

## Competing interests

The author(s) declare that they have no competing interests.

## Authors' contributions

HSS : Conceived the idea of above technique, conducted, coordinated the study and performed the surgeries.

TD : Performed the surgeries and helped in documentation of cases

HKR : Performed the surgeries and helped in documentation of cases

PS : Compiled the data and helped in the preparation of the manuscript

All authors read and approved the final manuscript.

## Pre-publication history

The pre-publication history for this paper can be accessed here:
